# Uncovering Frailty in Burning Mouth Syndrome: Insights From a SUNFRAIL+ Based Multidimensional Assessment

**DOI:** 10.1111/joor.70191

**Published:** 2026-03-26

**Authors:** Federica Canfora, Giulia Ottaviani, Antonietta Argiuolo, Alessandra Giorgiutti, Guido Iaccarino, Katia Rupel, Sabina Saccomanno, Vincenzo De Luca, Michele Virgolesi, Elizabeth Moloney, Rónán O'Caoimh, Giuseppe Liotta, Michele Davide Mignogna, Maddalena Illario, Daniela Adamo

**Affiliations:** ^1^ Department of Neurosciences, Reproductive Sciences and Odontostomatology University of Naples “Federico II” Naples Italy; ^2^ Department of Surgical, Medical and Health Sciences University of Trieste Trieste Italy; ^3^ Departmental Program of Clinical Psychopathology Federico II University Hospital Naples Italy; ^4^ Department of Clinical Medicine and Surgery Federico II University of Naples Naples Italy; ^5^ Department of Life Science, Health, and Health Professions Link Campus University Rome Italy; ^6^ Department of Public Health Federico II University of Naples Naples Italy; ^7^ Health Service Executive of Ireland, National Oral Care Guideline (Supporting Smiles) Development Group and Mercy University Hospital Cork City Ireland; ^8^ Health Research Board Clinical Research Facility, University College Cork Mercy University Hospital Cork City Ireland; ^9^ Department of Biomedicine and Prevention University of Rome “Tor Vergata” Rome Italy

**Keywords:** aging, bio‐psycho‐social model of frailty, Burning Mouth Syndrome, frailty, health promotion, multidimensional assessment, screening

## Abstract

**Background:**

Frailty reflects an age‐associated progressive decline in physiological reserve, increasing vulnerability to adverse outcomes. Burning Mouth Syndrome (BMS) is a chronic idiopathic orofacial pain disorder commonly seen in older individuals, yet its relationship with frailty remains uninvestigated.

**Objectives:**

This study aims to assess frailty in BMS patients using the *SUNFRAIL+* multidimensional screening tool, which facilitates early detection of bio‐psycho‐social risk factors for frailty in community‐dwelling older adults.

**Methods:**

This multi‐centre case–control study included 104 adults aged ≥ 65 years (52 with BMS and 52 age and sex matched controls). The *SUNFRAIL+* tool was used to identify risks and trigger further single‐domain evaluation with the Tool for Adherence to Therapies (TAS), Mini‐Nutritional Assessment (MNA), Mediterranean Diet Adherence Screener (PREDIMED), Short Physical Performance Battery (SPPB), Timed Up and Go test (TUG), Quick Mild Cognitive Impairment (Q*mci*) screen, General Practitioner Assessment of Cognition (GPCOG), Geriatric Depression Scale (GDS), Social Provisions Scale (SPS), socio‐economic conditions self‐assessment questionnaire (MUSE) and 12‐item Short Form Health Survey (SF‐12).

**Results:**

Patients with BMS showed significantly higher levels of frailty, characterized by higher rates of polypharmacy (76.9% vs. 30.8%), reduced mobility (61.5% vs. 42.3%; *p* > 0.05), greater cognitive impairment (Q*mci* 56.8 vs. 75.5), more self‐reported depressive symptoms (GDS: 7.5 vs. 4), less social support (SPS: 30 vs. 36), and lower quality of life (SF‐12; *p* < 0.05).

**Conclusions:**

Older adults with BMS have a greater proportion of clinical markers of frailty. The *SUNFRAIL+* model is effective in revealing hidden vulnerabilities, supporting early, multidisciplinary intervention in this cohort.

## Graphical abstract

Burning Mouth Syndrome patients showed a markedly higher frailty alert rate than controls using the SUNFRAIL+ multidimensional screening tool, with increased polypharmacy, cognitive decline, reduced mobility and depressive symptoms. These findings suggest that BMS is associated with a broader frailty profile requiring integrated physical, cognitive and psychosocial assessment.

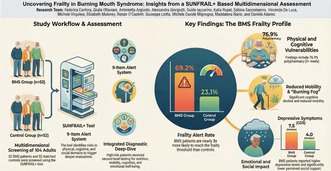

## 1 Introduction

Burning Mouth Syndrome (BMS) is a chronic idiopathic pain disorder characterized by persistent intraoral burning sensations without visible mucosal lesions [1]. Symptoms are usually bilateral and must persist for at least 2 h per day over a period of 4–6 months [1]. Patients often report additional dysesthetic and perceptual disturbances, such as xerostomia, dysgeusia, globus sensation, oral dysmorphism and halitophobia, which further complicate the clinical picture [2].

Although its aetiology remains uncertain, BMS is increasingly recognized as a chronic pain condition with both neuropathic and nociplastic features, involving altered nociceptive processing without tissue damage [3]. Biological, psychological and social factors, including depression, anxiety, cognitive impairment, sleep disturbances and social isolation, contribute to symptom persistence and overlap with domains affected by frailty [4]. BMS also significantly impacts quality of life (QoL), particularly in older adults, affecting approximately 5% of this cohort and occurs between 2.5 and 7 times more commonly in women than men, especially in postmenopausal females [5]. The average age at diagnosis is rising in parallel with population aging, reinforcing the view that BMS may act as a potential marker of age‐related vulnerability rather than solely a local oral disorder [6].

**FIGURE 1:**
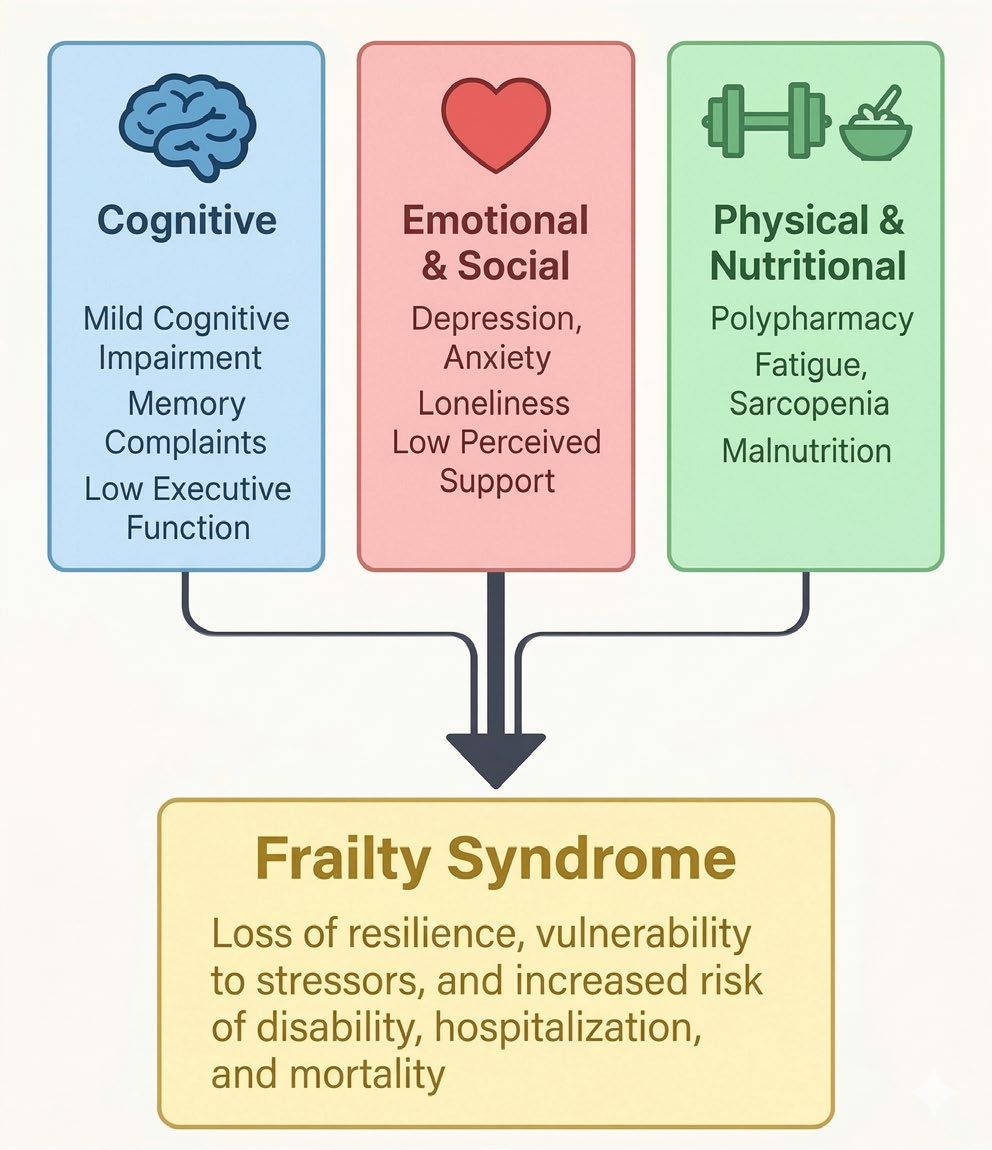
Frailty as a multidimensional syndrome in aging.

Older adults with BMS frequently present with multiple comorbidities and psychological factors that emerge as crucial determinants of clinical outcomes [7]. Frailty is an age‐associated multidimensional syndrome reflecting the cumulative decline of physiological systems, leading to increased vulnerability to stressors and higher risks of falls, disability, hospitalization and mortality (Figure 1) [8]. Frail older adults present with lower QoL, greater dependence on caregivers and increased healthcare needs. The bio‐psycho‐social paradigm of frailty integrates more functional areas (physical, psychological and social) than other models and helps stratify the risk of adverse outcomes to support clinical practice. Medical, environmental, educational, economic and psychological aspects impact older adults' health and independence and require a more holistic approach [9].

Despite growing awareness of the importance of frailty screening, no prior study has investigated frailty in individuals with BMS. Given the chronic nature of the disorder and its prevalence in older adults, exploring this relationship may aid in the early identification of vulnerable patients and foster better integrated care for this population. The *SUNFRAIL+* study is a multicentric observational study aimed at early identification of frailty in community‐dwelling older adults to prevent functional decline and promote personalized interventions. The *SUNFRAIL* tool, a nine‐item questionnaire assessing physical, cognitive and social domains [10], provides a multidimensional assessment of older adults by linking each item of the tool with an in‐depth assessment of the individual bio‐psycho‐social domains of frailty. The *SUNFRAIL+* multidimensional screening approach includes a digital and integrated system covering nine specific areas: polypharmacy, nutrition, physical performance, fall risk, cognitive function, emotional well‐being, social support, economic hardship and access to healthcare. This approach allows for a detailed profile of frailty and to guide personalized interventions [11].

This study aimed to evaluate frailty and its related domains in older adults with BMS using the *SUNFRAIL+* multidimensional assessment approach. Through a matched case–control design conducted across two Italian centres, patients with BMS were compared with age and sex‐matched controls to delineate frailty profiles and identify domains of vulnerability. By integrating clinical, functional, psychological and social parameters, the study provides insight into the multidimensional nature of BMS and supports more comprehensive, personalized and multidisciplinary care strategies.

## 2 Material and Methods

### 2.1 Study Design

This study was conducted as a multicentre case–control investigation involving the recruitment of older adult participants from two Italian centres: the Oral Medicine and Pathology Unit at the Clinic of Maxillofacial Surgery and Odontostomatology (Maggiore Hospital, Trieste, Italy) and the Oral Medicine Unit, together with other outpatient clinics, of the Federico II University of Naples (Naples, Italy).

Participants were recruited at both centres between February 2024 and June 2025 according to the same predefined inclusion and exclusion criteria. Participants recruited in Naples were identified from a subgroup of older adults enrolled in the SUNFRAIL+ study (December 2022 to June 2025), while recruitment in Trieste was conducted among patients attending the Oral Medicine and Pathology Unit during routine clinical visits.

Socio‐demographic characteristics, education level and Body Mass Index (BMI) were recorded for each enrolled patient. All participants underwent a multidimensional assessment using the *SUNFRAIL+* questionnaire, which is specifically designed to evaluate various domains of frailty in older adults.

The study was carried out in full compliance with current regulations governing clinical research. No data were collected without first providing participants with comprehensive information about the study and obtaining their written informed consent. All personnel involved in the research were responsible for taking all reasonable measures to ensure the ethical conduct of the study, strict adherence to the research protocol and the integrity and validity of the data collected.

Ethical approval for the study was obtained before patient recruitment from the Ethics Committee of the University “Federico II” Azienda Ospedaliera di Rilievo Nazionale “A. Cardarelli” (protocol no. 284/22) and from the Ethics Committee of the University of Trieste (protocol no. 02/2024), in accordance with current regulations concerning research involving human subjects and the principles of the Declaration of Helsinki.

The study protocol was prospectively registered on ClinicalTrials.gov on 9 December 2022 (registration number: NCT05646472) before participant recruitment began.

The reporting of this study conforms to the STROBE statement.

### 2.2 Selection and Characteristics of Participants

Participants were selected according to predefined inclusion and exclusion criteria to ensure a homogeneous and comparable study population, while minimizing confounding factors. For the BMS group, patients with a confirmed diagnosis of BMS, according to the International Classification of Orofacial Pain (ICOP) 2020 criteria, following a comprehensive clinical assessment performed by oral medicine specialists were included [1]. Eligible individuals were recruited during routine visits to participating centers, with both groups required to meet the inclusion and exclusion criteria defined by the study protocol. Inclusion criteria were: age ≥ 65 years; residence in the community (i.e., not institutionalized); access to general healthcare services at the recruiting centers; and provision of signed informed consent. Exclusion criteria included: age < 65 years; residing in long‐term care or institutional facilities; diagnosis of frailty or disability already in place; current receipt of home‐based care services or refusal to provide informed consent. The Control group consisted of age and sex‐matched individuals without a history of BMS, fulfilling the same inclusion and exclusion criteria, except for the presence of BMS symptoms.

### 2.3 Data Collection

Data were collected using the *SUNFRAIL+* questionnaire, a validated multidimensional tool for the identification of frailty in older adults, developed and validated in Italy [12]. Details on the data collection have been published elsewhere [12] but in summary, clinical history, including the presence of comorbidities and current pharmacological treatments, was obtained from electronic medical records and confirmed during interviews. Data collection took place at baseline and was performed by trained professionals during one‐on‐one interviews conducted in a reserved clinical setting. Prior to study initiation, all members of the research team underwent a standardized training focused on Burning Mouth Syndrome, including diagnostic criteria, clinical features and the administration of study‐specific assessment tools. The training also included calibration sessions on the standardized administration of questionnaires and clinical tests to ensure methodological consistency and reduce inter‐operator variability across investigators. All data were pseudo‐anonymized by assigning each participant a unique identification code. The *SUNFRAIL+* tool, a structured 9‐item questionnaire (Table 1) specifically designed to identify early signs of multidimensional frailty in older adults. The tool investigates three broad domains: physical (polypharmacy, mobility, falls and sensory issues), cognitive/psychological (emotional health) and socio‐economic (social support, income, housing) [13]. Each alert contributes one point to the overall score, yielding a total that can range from 0 to 9. Following prior validation studies in Italian community‐dwelling older populations, including the article by De Luca et al. [14], a cut‐off score of three or more indicates that an individual is frail. This threshold reflects the presence of at least three risk factors distributed across different domains and correlates with increased clinical vulnerability and worse health outcomes [14].

**TABLE 1:** Sociodemographic characteristics, education level and SUNFRAIL first‐level screening results comparing patients with Burning Mouth Syndrome (BMS) and healthy controls.

Demographic variables	BMS (*n* = 52)	Controls (*n* = 52)	*p*
Gender	(*n*; %)	(*n*; %)	
F	45 (86.5%)	41 (78.9%)	0.30
M	7 (13.5%)	11 (21.1%)	
Age (in years)	Median [Q1–Q3] 73 [69–77.3]	Median [Q1–Q3] 75.5 [68.8–82]	0.24
Education (in years)	Median [Q1–Q3] 8 [5–13]	Median [Q1–Q3] 13 [5–13]	0.004*
SUNFRAIL	(*n*; %)	(*n*; %)	
Score ≥ 3	36 (69.2%)	12 (23.1%)	< 0.001*

SUNFRAIL DOMAIN checklist and domains				
Screening questions	Domain	Yes (*n*; %)	Yes (*n*; %)	
1. Do you take 5 or more medicines daily?	Polypharmacy PHYSICAL	40 (76.9%)	16 (30.8%)	< 0.001*
2. Have you experienced weight loss recently?	Nutrition PHYSICAL	10 (19.2%)	9 (17.3%)	0.8
3. Do you have difficulty walking or moving around during the last year?	Mobility PHYSICAL	32 (61.5%)	22 (42.3%)	0.05*
4. Have you been evaluated by your GP during the last year?	Access to/need for healthcare PHYSICAL	46 (88.5%)	36 (69.2%)	0.016*
5. Have you fallen 1 or more times during the last year?	Falls PHYSICAL	12 (23.1%)	22 (42.3%)	0.037*
6. Have you experienced memory decline during the last year?	Cognition PSYCHOLOGICAL	35 (67.3%)	16 (30.8%)	0.001*
7. Do you feel lonely most of the time?	Social isolation/Emotional well‐being SOCIAL	23 (44.2%)	11 (21.1%)	0.012*
8. In case of need, can you count on someone close to you?	*Social support* *SOCIAL*	41 (78.9%)	47 (90.4%)	0.103
9. Have you had any financial difficulty in facing dental care and health care costs during the last year	Socioeconomic status SOCIAL	8 (15.4%)	3 (5.8%)	0.111

*Note:* Data are expressed as median and interquartile range (IQR) for continuous variables, and as absolute numbers and percentages for categorical variables. SUNFRAIL data are reported as the number of participants responding “Yes” for each item, over the total number of subjects per group. A significant difference between the percentages was measured by the Pearson Chi‐Squared test. A significance difference between the medians were measured by the Mann–Whitney *U* test.

Abbreviations: BMS, Burning Mouth Syndrome; GP, General Practitioner; IQR, interquartile range.

* Significant *p* ≤ 0.05.

### 2.4 Second‐Level Tools Administered According to SUNFRAIL Items

Polypharmacy (Item 1) was further assessed using the Therapeutic Adherence Scale (TAS), a brief four‐item questionnaire designed to capture common behaviours related to medication use [15]. The tool assesses behaviours such as forgetting to take medications, self‐adjusting dosages, discontinuing treatment when feeling better, or skipping doses for other reasons. Each item is scored dichotomously, assigning 1 point for adherent and 0 points for non‐adherent behaviour, with a total score ranging from 0 to 4, and lower scores indicate poorer adherence. According to established criteria, participants scoring 0–2 were classified as non‐adherent, while those scoring 3–4 were considered adherent [15].

Nutrition (Item 2) was evaluated using two complementary tools: the Mediterranean Diet Adherence Screener (PREDIMED) and the Mini‐Nutritional Assessment‐Short Form (MNA‐SF) [16, 17]. Adherence to the Mediterranean diet was assessed using the PREDIMED questionnaire, a 14‐item dichotomous (yes/no) tool evaluating dietary habits. Total scores range from 0 to 14, with scores ≤ 5 indicating low adherence, 6–9 moderate adherence and ≥ 10 high adherence to the Mediterranean dietary pattern [16]. Nutritional status was assessed using the MNA‐SF, a validated six‐item screening tool addressing recent weight loss, appetite changes, mobility, psychological stress, neuropsychological conditions and body mass index (BMI). Following the SUNFRAIL+ protocol, MNA‐SF scores below 12 were classified as indicative of nutritional frailty, while scores ≥ 12 were considered non‐frail [17].

Mobility (Item 3) was evaluated using the Short Physical Performance Battery (SPPB), which includes three components: balance tests (side‐by‐side, semi‐tandem and tandem stance), a 4‐m walking speed test and the chair stand test (five repetitions). Each domain is scored from 0 to 4, for a total score from 0 (worst) to 12 (best performance) [18]. Higher scores indicate better physical function, while scores ≤ 9 suggest reduced mobility and increased frailty risk [18]. Access to healthcare (Item 4) was evaluated using the General Practitioner (GP) Visiting Checklist, a brief screening tool designed to detect irregularities in routine medical follow‐up. It includes items assessing the frequency of GP appointments, specialist consultations and adherence to recommended check‐ups [14]. If one or more items are answered positively (e.g., “I have not visited my GP in the past 12 months”), this indicates poor access or attendance at medical care. The checklist does not generate a cumulative score but classifies dichotomists with adequate or inadequate healthcare access/attendance.

The risk of falls (Item 5) was assessed using two complementary tools. First, the Aged‐Friendly Environment Assessment Tool (AFEAT) was employed to evaluate how supportive and safe the participant's living environment is for older adults [19]. It consists of 10 items scored on a 5‐point Likert scale, resulting in a total score ranging from 10 to 50, where higher scores indicate a more age‐friendly and safer environment (e.g., adequate lighting, accessible spaces, absence of obstacles) [19]. Second, falls risk was measured using the Timed Up and Go Test (TUG) functional mobility test [20]. Participants were asked to stand up from a chair, walk three meters at a comfortable pace, turn around, walk back and sit down again. The total time, measured in seconds, reflects lower limb strength, balance and gait speed [20]. Shorter times correspond to better mobility and lower fall risk, while times > 13.5 s are generally considered indicative of an increased risk of falls in community‐dwelling adults [20].

Cognitive function (Item 6) was evaluated using two complementary instruments. The Quick Mild Cognitive Impairment (Q*mci*) screen is a brief, validated tool that assesses six cognitive domains: orientation, registration, clock drawing, delayed recall, verbal fluency and logical memory [21]. The total score ranges from 0 to 100, with lower scores indicating greater cognitive impairment. A score of ≤ 49.4 is considered indicative of cognitive decline when using the Italian version [21]. The General Practitioner Assessment of Cognition (GPCOG) includes two sections: a patient cognitive test (maximum score = 9) and an informant interview (maximum score = 6) [22]. A perfect score of 9 on the patient section indicates normal cognition. Scores between 5 and 8 require the informant section to be considered. If the informant score is ≤ 3, cognitive impairment is likely; scores between 4 and 6 suggest mild impairment, warranting further follow‐up [22].

Emotional well‐being (Item 7) was assessed using the Geriatric Depression Scale‐Short Form (GDS‐15), a 15‐item questionnaire widely used to detect depressive symptoms in older adults [23]. Each item is scored dichotomously (Yes/No), yielding a total score from 0 to 15. Scores ≤ 5 indicate that depression is unlikely, scores between 6 and 9 suggest possible mild depression, and scores ≥ 10 are indicative of probable clinically relevant depression [23].

Perceived social support (Item 8) was evaluated using the Social Provisions Scale (SPS), a 10‐item instrument designed to measure six key dimensions of supportive relationships: attachment, social integration, guidance, reassurance of worth, reliable alliance and opportunity for nurturance. Items are rated on a 4‐point Likert scale ranging from 1 (“strongly disagree”) to 4 (“strongly agree”), with higher total scores reflecting stronger perceived social support [24]. Socioeconomic vulnerability (Item 9) was assessed using the Multidimensional Socio‐Economic Self‐Assessment (MUSE) questionnaire, which explores several key dimensions of socioeconomic status, including perceived income adequacy, housing quality, access to essential services and financial security [14, 25]. Although the original MUSE tool does not provide a standardized cumulative score, in the present study a composite numerical score ranging from 0 to 10 was calculated by summing item responses in order to quantify overall socioeconomic vulnerability. Lower scores reflect greater levels of perceived socioeconomic disadvantage. This operationalization allowed for inclusion in the statistical analysis and facilitated comparisons between groups [25].

Health‐related quality of life (HRQoL) was assessed using the Short Form Health Survey (SF‐12 v1), the validated Italian version [26, 27]. The instrument provides two summary indices: the Physical Component Summary (PCS) and the Mental Component Summary (MCS), obtained by applying the weighted scoring algorithm for each of the 12 items [27].

Therefore, PCS and MCS values are expressed as unstandardized continuous scores (range approximately 0–200), where higher values indicate better perceived physical and mental health. The comparison between groups was performed on these raw values, which maintain the same statistical meaning as standardized ones [26].

All questionnaires are described in detail in the Appendix S1, and their correspondence with SUNFRAIL items is reported in Table 2.

**TABLE 2:** Second‐level assessment tools and scoring criteria according to the SUNFRAIL+ protocol.

Item (*n*.)	Domain	Test	Description	Score (cut‐off)
1	Polypharmacy	Therapeutic Adherence Scale (TAS)	4 dichotomous items assessing medication‐taking behaviours (forgetting, skipping, stopping, reducing).	0–2 nonadherent 3–4 adherent
2	Nutrition	Assessment of Adherence to the Mediterranean Diet (PREDIMED) Mini Nutritional Assessment‐Short Form (MNA‐SF)	14 yes/no items assessing adherence to the Mediterranean diet. 6 items on appetite, weight, loss, mobility, stress and body mass index	PREDIMED: ≤ 5 poor adherence 6–9 medium ≥ 10 good MNA‐SF < 12 nutritional frailty
3	Mobility	Short Physical Performance Battery (SPPB)	Three subtests: balance, 4 m gait speed and 5‐time chair stand	0 (worst) –12 (best performance) Total score ≤ 9 reduced mobility and increased frailty risk
4	Access to healthcare (Alert is negative)	GP visiting checklist	Checklist assessing regularity of GP or specialist visits.	≥ 1 positive item = poor healthcare adherence
5	Falls	Aged‐Friendly Environment Assessment Tool (AFEAT); Timed Up and Go Test (TUG)	10 items evaluating environmental safety and accessibility. Time (in seconds) to stand, walk 3 m, return and sit.	AFEAT: 10 (low)–50 Higher = safer/more age‐friendly environment TUG: less time = better performance
6	Cognition	Quick Mild Cognitive Impairment (Q*mci*) screen; General Practitioner Assessment of Cognition (GPCOG) score	6 subtests: orientation, immediate and delayed recall, clock drawing, verbal fluency, logical memory 2‐part test: patient section (0–9) and optional informant section (0–6).	0–100; ≤ 49.4 = cognitive deficit 50–62 = MCI; > 62 = normal QMCI Part A = 9 → normal; Part A 5–8 + B ≤ 3 → cognitive deficit; Part A 5–8 + B 4–6 → MCI (monitor over time)
7	Social isolation	Geriatric Depression Scale (GDS)	15 yes/no items assessing mood and depressive symptoms in older adults.	≤ 5 = normal 6–9 = mild depression; ≥ 10 = moderate–severe
8	Social support (Alert is negative)	Social Provision Scale (SPS)	10 items on attachment, social integration, guidance, reassurance and reliable alliance.	Higher score = stronger perceived social support
9	Socioeconomic status	Socio‐economic conditions self‐assessment questionnaire (MUSE)	Quantitative tool evaluating perceived economic stability, access to essential services, housing conditions and financial security.	0–10; lower scores indicate poorer socio‐economic conditions
10	Quality of life	Short Form‐12 Health Survey (SF‐12, v.1)	12‐item tool evaluating eight domains of health: physical functioning, role limitations (physical and emotional), bodily pain, general health, vitality, social functioning and mental health. Provides two composite indices: Physical Component Summary (PCS) and Mental Component Summary (MCS).	Standardized scores (mean = 50, SD = 10). Higher values indicate better physical and mental quality of life. No absolute cut‐off; lower scores reflect poorer perceived health status.

### 2.5 Statistical Analysis

Statistical analyses were conducted using Jamovi software (version 2.6; The Jamovi Project, 2024). The Shapiro–Wilk test revealed that most variables violated the assumption of normality; therefore, non‐parametric methods were employed. Between‐group comparisons for continuous variables were assessed using the Mann–Whitney U test, while categorical variables were compared using Pearson's Chi‐squared test. Two‐tailed *p*‐values ≤ 0.05 were considered statistically significant. A post hoc power analysis was performed using G*Power (version 3.1.9.7) [28] to estimate the statistical power of the conducted tests. For the Mann–Whitney U tests, observed effect sizes (*r*) ranged from 0.04 to 0.78 (mean = 0.32), based on a sample size of 52 participants per group, applying an alpha level of 0.05, and assuming a two‐tailed hypothesis. The corresponding statistical power (1 − *β*) ranged from 0.05 to 0.97 (mean = 0.44). For the Chi‐squared tests, effect sizes (φ the Phi) varied from 0.03 to 0.46 (mean = 0.15), with observed power ranging between 0.06 and 0.99 (mean = 0.60).

## 3 Results

The final sample included a total of 104 participants, 52 diagnosed with BMS and 52 matched healthy controls (Table 1). The two groups did not differ significantly in terms of sex: 45 women with BMS versus 41 controls; *p* = 0.30 or age in years (Median and interquartile range [IQR]: 73 [8.25] vs. 75.5 [13.3]; *p* = 0.24). However, those with BMS reported statistically significantly less time in formal education (Median [IQR] years: 8 [8] vs. 13 [8] years; *p* < 0.05).

Screening using the *SUNFRAIL+* first‐level tool revealed that patients with BMS were significantly more likely to reach the alert threshold for frailty, with 69.2% of cases scoring ≥ 3 compared to 23.1% of healthy controls (*p* < 0.001) (Table 1). Examining domains triggered on the *SUNFRAIL+* questionnaire, these were significant for the physical domain; patients with BMS more often reported daily polypharmacy (76.9% vs. 30.8%, respectively; *p* < 0.001), reduced mobility over the past year (61.5% vs. 42.3%; *p* = 0.05), and a higher proportion of patients adhering to scheduled medical visits within the one‐year timeframe (88.5% vs. 69.2%, respectively; *p* = 0.016). A lower proportion of patients with BMS reported experiencing falls compared to controls (23.1% vs. 42.3%; *p* = 0.037). In the psychosocial domain, those with BMS more frequently reported subjective memory complaints (67.3% vs. 30.8%; *p* = 0.001) and feelings of loneliness (44.2% vs. 21.1%; *p* = 0.012). Medication adherence evaluated through the TAS (Table 3) showed that BMS patients more frequently reported forgetting to take their medications (40.4% vs. 15.4%; *p* = 0.004) and skipping doses (65.4% vs. 88.5%; *p* = 0.005). There was no difference in stopping medication when feeling better (86.5% vs. 92.3%; *p* = 0.26) or dose reduction (82.7% vs. 94.2%; *p* = 0.06). Overall, patients with BMS had a slightly lower medication adherence compared to healthy controls (Median [IQR]: 3 [2] vs. 4 [1], respectively), though both groups may be considered adherent. Those with BMS reported more psychological stress or recent acute disease (55.8% vs. 21.2%; *p* < 0.001) and had significantly higher neuropsychological problem scores (32.69% vs. 7.69%; *p* = 0.001).

**TABLE 3:** Analysis of medication adherence (TAS), dietary habits (PREDIMED) and nutritional status (MNA‐SF) in BMS patients and healthy controls.

TAS	BMS	Controls	*p*
	(*n*, %)	(*n*, %)	
Forget taking medicine	21 (40.38%)	8 (15.38%)	0.004*
Stop taking medicine when feeling better	45 (86.54%)	48 (92.31%)	0.339
Skip a dose	34 (65.38%)	46 (88.46%)	0.005*
Dose reduction	43 (82.69%)	49 (94.23%)	0.066

	Median [Q1–Q3]	Median [Q1–Q3]	
PREDIMED	8 [7–9]	9 [8–10]	0.01*
Mini Nutritional Assessment Short Form (MNA‐SF)			
Dietary assessment	(*n*, %)	(*n*, %)	
Appetite loss (< 2)	16 (30.8%)	10 (19.23%)	0.174
General assessment			
Reduced mobility (< 2)	6 (11.54%)	8 (15.38%)	0.566
Psychological stress or acute disease (yes)	29 (55.77%)	11 (21.15%)	< 0.001*
Neuropsychological problems (< 2)	17 (32.69%)	4 (7.69%)	0.001*
Anthropometric assessment			
BMI (19 ≤ BMI < 23)	25 (48.08%)	43 (82.69%)	< 0.001*
Calf circumference (> 31 cm)	7 (13.46%)	2 (3.85%)	0.08
Weight loss (yes)	16 (30.8%)	17 (32.69%)	0.833

	Median [Q1–Q3]	Median [Q1–Q3]	
MNA total	11 [10.8–13]	12 [11–13]	0.484

*Note:* Data are presented as the median and interquartile range for scale totals, while as frequencies and percentages for each item, relative to the total number of subjects in each group. A significant difference between the percentages was measured by the Pearson Chi‐Squared test. A significant difference between the medians was tested using the Mann–Whitney *U* test. Data are expressed as median and interquartile range (IQR), unless otherwise specified. For the item “Appetite loss” values represent the number and percentage of participants reporting a severe or moderate appetite loss in the last 3 months. For the item “Reduced mobility” values represent the number and percentage of participants reporting that their mobility was reduced to their home. For the item “Psychological stress or acute disease,” values represent the number and percentage of participants responding “Yes.” For the item “Neuropsychological problems” values represent the number and percentage of participants reporting severe or moderate dementia. For the item “BMI” values represent the number and percentage of participants reporting a BMI within the normal weight. For the item “Calf circumference,” values indicate the number and percentage of participants with a circumference > 31 cm. For the item “Weight loss” values represent the number and percentage of participants reporting some weight loss. Between‐group differences for categorical variables were tested using Pearson's Chi‐squared test. Differences in medians were assessed using the Mann–Whitney *U* test.

Abbreviations: BMI, body mass index; BMS, Burning Mouth Syndrome; MNA‐SF, Mini Nutritional Assessment‐Short Form; PREDIMED, Prevención con Dieta Mediterránea; SD, standard deviation; TAS, Therapeutic Adherence Scale.

* Significant *p* ≤ 0.05.

**FIGURE 2:**
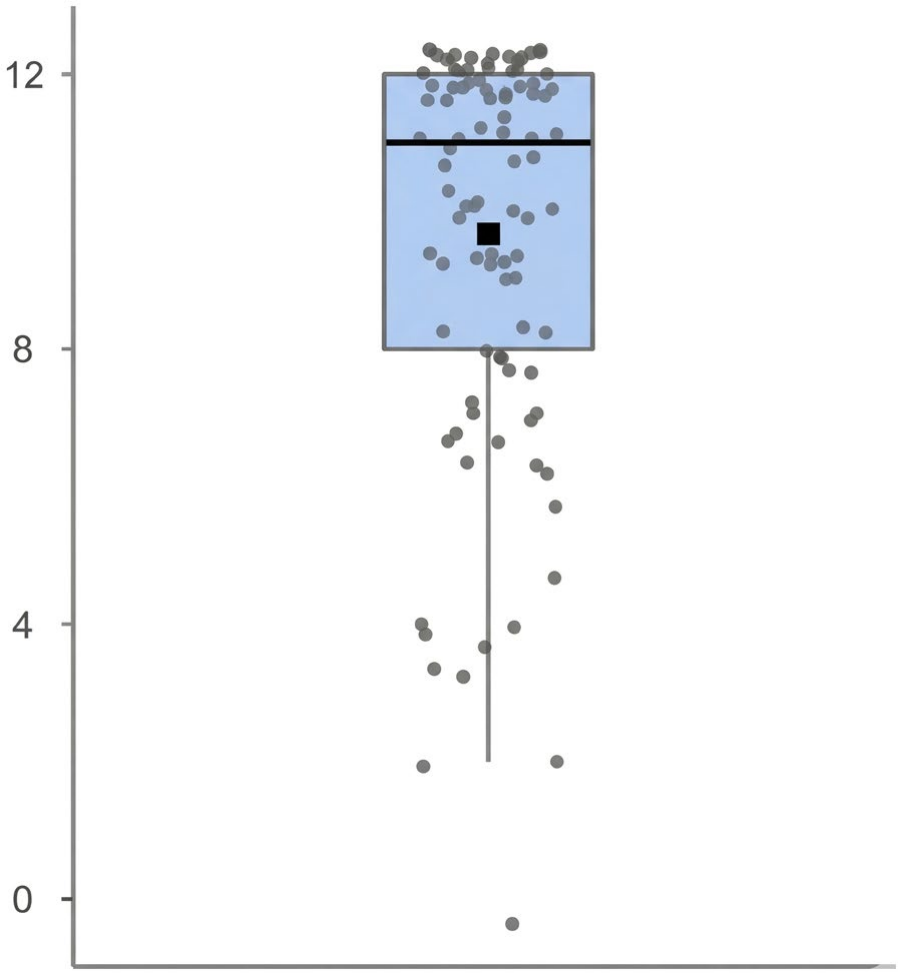
Box‐plot of SPPB total score distribution.

Dietary adherence and nutritional status are detailed in Table 3. Patients with BMS also had lower adherence to the Mediterranean diet (PREDIMED score: 8 [2] vs. 9 [2]; *p* = 0.01). Nutritional assessment did not differ significantly between groups (MNA‐SF total scores: 11 [2.25] vs. 12 [2]; *p* = 0.484). Additionally, they presented with significantly lower BMI scores (48.08% vs. 82.69%; *p* < 0.001), meaning that BMS patients had more likelihood to be underweight or overweight. No significant group differences were observed for appetite loss (30.8% vs. 19.23%; *p* = 0.174), reduced mobility (11.54% vs. 15.38%; *p* = 0.566), weight loss (30.8% vs. 32.69%; *p* = 0.833), or calf circumference greater than 31 cm (13.46% vs. 3.85%; *p* = 0.08). The evaluation of physical performance using the SPPB revealed significantly more frequent impaired scores in BMS patients across all subcomponents (Table 4). A score in Balance test below 4 (normal performance) was more frequent in the BMS group (40.08% vs. 25%), so as the Gait speed test (51.92% vs. 26.92%) and the Chair stand test (71.15% vs. 23.1%). These impairments contributed to significantly reduced overall SPPB total scores in the group with BMS (9 [IQR 4] vs. 12 [IQR 1]; *p* < 0.001; Figure 2). No significant group differences were found in environmental safety, as assessed by the AFEAT, with similar total scores (36 [IQR 5] vs. 37 [IQR 6]; *p* = 0.42). Similarly, performance on the TUG, which evaluates functional mobility and risk of falls, did not differ significantly between groups (12.5 [IQR 3.2] vs. 12.1 [IQR 2.8]; *p* = 0.61).

**TABLE 4:** Physical performance and environmental safety: Results of the Short Physical Performance Battery (SPPB), Aged‐Friendly Environment Assessment Tool (AFEAT) and Timed Up and Go Test (TUG) and cognitive performance assessed by the General Practitioner Assessment of Cognition (GPCOG) and Quick Mild Cognitive Impairment screen (Q*mci*).

SPPB	BMS	Controls	*p*
	(*n*, %)	(*n*, %)	
Balance test	25 (48.08%)	13 (25%)	0.015*
Gait speed test	27 (51.92%)	14 (26.92%)	0.009*
Chair stand test	37 (71.15%)	12 (23.1%)	< 0.001*
*SPPB total*	9 [7–11]	12 [11–12]	< 0.001*

AFEAT	Median [Q1–Q3]	Median [Q1–Q3]	p
Total score	36 [24–38]	37 [26–40]	0.42

TUG	Median [Q1–Q3]	Median [Q1–Q3]	p
Total score	12.5 [9–13]	12.1 [10–13.5]	0.61
GPCog total	6 [4–8]	8 [6.75–8]	0.013*
Q*mci* screen			
Orientation	10 [10–10]	10 [10–10]	0.14
Immediate recall	4 [3–5]	5 [4–5]	< 0.001*
Delayed recall	16 [12–20]	16 [15–20]	0.53
Logical memory	10 [6–12]	18 [12–20]	< 0.001*
Clock drawing	9.50 [3–15]	15 [14–15]	< 0.001*
Verbal fluency	7 [6–9]	15.5 [11–18]	< 0.001*
Q*mci* screen *total*	56.80 [42.75–62.50]	75.5 [69.75–86.25]	< 0.001*

*Note:* For SPPB item, data are expressed as frequencies and percentage of impaired performance (a score below 4), while for other variables median and interquartile range (IQR) are reported. A significant difference between medians was measured by the Mann–Whitney U test, while between‐group differences for categorical variables were tested using Pearson's Chi‐squared test.

Abbreviations: AFEAT, Aged‐Friendly Environment Assessment Tool; BMS, Burning Mouth Syndrome; GPCog, General Practitioner assessment of Cognition; IQR, Interquartile Range; Q*mci*, Quick Mild Cognitive Impairment; SPPB, Short Physical Performance Battery; TUG, Timed Up and Go Test.

* Significant *p* ≤ 0.05.

**FIGURE 3:**
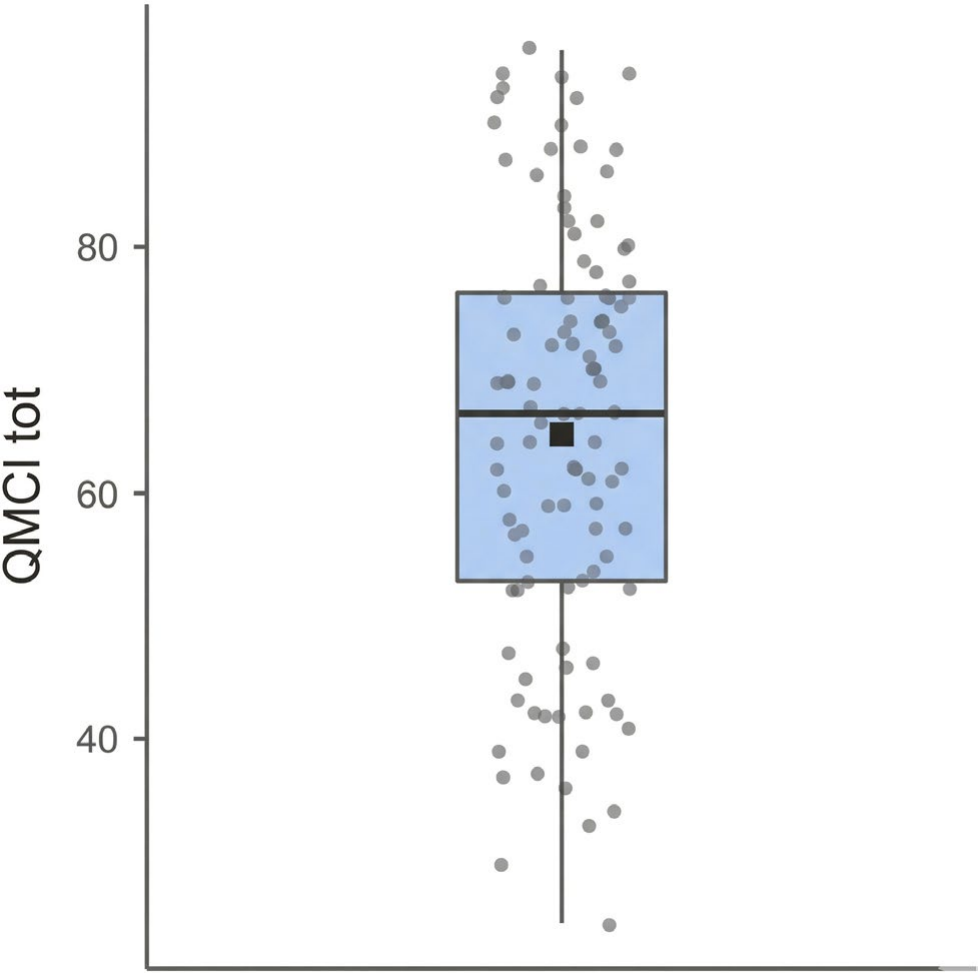
Box‐plot of QMCI total score distribution.

The analysis of cognitive tests in our sample (Table 4) showed that patients with BMS had lower GPCOG total scores compared to controls with respect to (median [IQR]: 6 [4] vs. 8 [1.25]; *p* = 0.013). Similarly, Q*mci* screen total scores were lower in the group with BMS (56.8 [19.8]) than the controls (75.5 [16.5]; *p* < 0.001) (Figure 3). When exploring specific cognitive domains using the Q*mci* screen, no significant differences emerged in orientation or delayed recall tasks (*p* = 0.14 and *p* = 0.53, respectively). However, participants with BMS performed significantly worse on immediate recall (4 [2] vs. 5 [1]; *p* < 0.001), logical memory (10 [6] vs. 18 [8]; *p* < 0.001), clock drawing (9.5 [12] vs. 15 [1]; *p* < 0.001) and verbal fluency (7 [3] vs. 15.5 [7]; *p* < 0.001).

Emotional well‐being assessed using the GDS (Table 5) showed that those with BMS reported significantly more depressive symptoms than healthy controls, as reflected in both the total GDS score (median [IQR]: 7.5 [5] vs. 4 [4.25]; *p* < 0.001) and in the frequency of several individual items. Specifically, patients with BMS were statically significantly more likely to report low life satisfaction (63.46% vs. 23.08%; *p* < 0.001), feelings of emptiness (48.08% vs. 7.69%; *p* < 0.001), fear that something bad might happen (55.77% vs. 28.85%; *p* = 0.005), reduced happiness (63.46% vs. 28.85%; *p* < 0.001), feelings of worthlessness (17.31% vs. 7.69%; *p* = 0.026), hopelessness (26.92% vs. 1.92%; *p* < 0.001) and subjective memory problems (36.54% vs. 7.69%; *p* < 0.001). They also reported lower energy (25% vs. 50%; *p* = 0.008) and vitality (61.54% vs. 42.31%; *p* = 0.05). While both groups showed similar rates for certain symptoms such as boredom (*p* = 0.45), inferiority (*p* = 0.65) and loss of interest (*p* = 0.33), patients BMS were more likely to endorse feeling isolated (44.23% vs. 63.46%; *p* = 0.049) and less likely to describe themselves as being in a good mood (51.92% vs. 32.69%; *p* = 0.047).

**TABLE 5:** Comparative analysis of depressive symptoms (GDS‐15), perceived social support (SPS), socioeconomic vulnerability (MUSE) and health‐related quality of life (SF‐12) in BMS patients and healthy controls.

*GDS*	BMS (*n*, %)	Controls (*n*, %)	*p*
Life satisfaction	33 (63.46%)	12 (23.08%)	< 0.001*
Loss of interests	23 (44.23%)	28 (53.85%)	0.33
Emptiness	25 (48.08%)	4 (7.69%)	< 0.001*
Boredom	11 (21.15%)	8 (15.38%)	0.45
Good mood	27 (51.92%)	17 (32.69%)	0.047*
Fear	29 (55.77%)	15 (28.85%)	0.005*
Happiness	33 (63.46%)	15 (28.85%)	< 0.001*
Helplessness	19 (36.54%)	37 (71.15%)	< 0.001*
Isolation	23 (44.23%)	33 (63.46%)	0.049*
Memory problems	19 (36.54%)	4 (7.69%)	< 0.001*
Vitality	32 (61.54%)	22 (42.31%)	0.05*
Worthlessness	9 (17.31%)	4 (7.69%)	0.026*
Energy	13 (25%)	26 (50%)	0.008*
Hopelessness	14 (26.92%)	1 (1.92%)	< 0.001*
Inferiority	2 (3.85%)	3 (5.77%)	0.65

	Median [Q1–Q3]	Median [Q1–Q3]	*p*
GDS total	7.5 [4–9]	4 [1.75–6]	< 0.001*

SPS	BMS (Median [Q1–Q3])	Controls (Median [Q1–Q3])	*p*
Reliable alliance	7 [6–8]	8 [7–8]	< 0.001*
Social integration	5 [4–6]	7 [5.75–8]	< 0.001*
Attachment	7 [6–8]	7.5 [6–8]	0.007*
Guidance	7 [6–8]	8 [6–8]	0.08
Reassurance of worth value	5 [4–6]	7 [6–8]	< 0.001*
SPS total	30 [28–33.25]	36 [32.75–39]	< 0.001*

MUSE	BMS (Median [Q1–Q3])	Controls (Median [Q1–Q3])	*p*
Total score	5 [4–8]	5 [4–9]	0.77

SF‐12	BMS (Median [Q1–Q3])	Controls (Median [Q1–Q3])	*p*
Physical component summary (PCS)	90.8 [72.3–105]	112 [69.5–119.4]	< 0.001*
Mental component summary (MCS)	135 [105.6–157.1]	171 [143.2–187.2]	< 0.001*
General health (GH)	25 [0–50]	50 [50–75]	< 0.001*
Physical functioning (PF)			
PF1 Physical Functioning: Moderate Activities	25 [18.8–31.3]	50 [25–50]	< 0.001*
PF2 Physical Functioning: Climbing Several Flights of Stairs	25 [25–50]	50 [25–50]	0.002*
Role physical (RP)			
RP1 Role Limitations due to Physical Health: Accomplished Less	0 [0–25]	25 [0–25]	0.022*
RP2 Role Limitations due to Physical Health: Limited in Kind of Work	0 [0–25]	25 [0–25]	0.001*
Role emotional (RE)			
RE1 Role Limitations due to Emotional Problems: Accomplished Less	25 [0–25]	25 [25–25]	0.004*
RE2 Role Limitations due to Emotional Problems: Less Careful than Usual	25 [0–25]	25 [25–25]	0.006*
Bodily pain (BP)	60 [40–60]	80 [60–100]	< 0.001*
Mental Health (MH)			
MH1 Mental Health: Felt Calm and Peaceful	40 [20–80]	80 [40–80]	< 0.001*
MH2 Felt Downhearted and Blue	60 [40–60]	80 [60–80]	< 0.001*
Itality (VT)	40 [20–40]	80 [40–80]	< 0.001*
Social functioning (SF)	75 [50–100]	100 [100–125]	< 0.001*

*Note:* Data are expressed as median (IQR) or percentage, as appropriate. Differences between groups were evaluated using the Pearson Chi‐squared test or the Mann–Whitney *U* test, as appropriate.

Abbreviations: BMS, Burning Mouth Syndrome; GDS, Geriatric Depression Scale; IQR, interquartile range; MUSE, Multidimensional Socio‐Economic Self‐Assessment; SD, standard deviation; SF‐12, 12‐Item Short Form Survey; SPS, Social Provisions Scale.

* Significant *p* ≤ 0.05.

Analysis of perceived social support, assessed using the SPS, showed that those with BMS had significantly lower scores compared to healthy controls (Table 5). Specifically, the total SPS score was significantly lower in the group with BMS (median [IQR]: 30 [5.25]) than in controls (36 [6.25]; *p* < 0.001). At the subscale level, BMS patients reported significantly lower scores in the domains of Reliable Alliance (7 [2] vs. 8 [1]; *p* < 0.001), Social Integration (5 [2] vs. 7 [2.25]; *p* < 0.001), Attachment (7 [2] vs. 7.5 [2]; *p* = 0.007) and Reassurance of Worth (5 [2] vs. 7 [2]; *p* < 0.001). No significant difference was observed in the Guidance domain (7 [2] vs. 8 [2]; *p* = 0.08). Regarding socioeconomic vulnerability, as assessed by the MUSE, no significant difference was found between patients with BMS and controls (median [IQR]: 5 [2] vs. 5 [1.5]; *p* = 0.77). HRQoL measured using the SF‐12 revealed significantly lower scores among patients with BMS compared to healthy controls across all measured domains (Table 5). Both the Physical Component Summary (PCS) and Mental Component Summary (MCS) scores were reduced in the BMS group (PCS: median [IQR] 90.8 [32.7] vs. 112 [22.9]; *p* < 0.001; MCS: 135 [51.5] vs. 171 [43.9]; *p* < 0.001). Within the physical functioning domain, patients with BMS scored significantly lower in both sub‐items: Physical Functioning for Moderate Activities (PF1;25 [12.5] vs. 50 [25]; *p* < 0.001) and Physical Functioning for intensive activities (PF2; 25 [25] vs. 50 [25]; *p* = 0.002). Similarly, role limitations due to physical problems were more frequently reported in BMS participants, with lower scores in both Role Physical Health components: less accomplishment (RP1 median: 0 [25] vs. 25 [25]; *p* = 0.022) and limited in kind of work (RP2: median0 [25] vs. 25 [25]; *p* = 0.001). Regarding emotional functioning, BMS patients also demonstrated greater impairment. Although the median values for RE1 (accomplished less) and RE2 (Less careful than usual) were the same (25 [25]), the narrower IQR among controls suggests significantly less variability (RE1: *p* = 0.004; RE2: *p* = 0.006).

Pain perception, assessed via the Bodily Pain domain, was also significantly different between groups, with those with BMS, as expected, reporting more severe pain (60 [20] vs. 80 [40]; *p* < 0.001). Likewise, participants in the BMS group had significantly lower scores in mental health‐related components such as MH1 (Felt Calm and Peaceful; 40 [60] vs. 80 [40]; *p* < 0.001) and MH2 (Felt Downhearted and Blue; 60 [20] vs. 80 [20]; *p* < 0.001). Finally, vitality and social functioning were both reduced in patients with BMS compared to controls. Those with BMS scored lower on vitality (40 [20] vs. 80 [40]; *p* < 0.001) and on the Social Functioning subscale (75 [50] vs. 100 [25]; *p* < 0.001).

## 4 Discussion

This study offers a comprehensive overview of the multidimensional frailty profile of older adults diagnosed with BMS, compared with age and sex matched controls. It reveals a statistically significantly higher prevalence of frailty related states and associations with physical, cognitive, psychosocial and nutritional frailty domains. These results underscore the systemic and complex nature of BMS and reinforce the need for an integrated and interdisciplinary clinical management approach [29]. The case–control design allowed for a robust comparison between individuals with BMS and matched controls, highlighting specific areas of vulnerability in the BMS population that should prompt the need for more detailed comprehensive geriatric (frailty) assessment among those with BMS.

Sociodemographic data revealed that patients with BMS had significantly lower educational attainment compared to controls. The observed difference in educational attainment between groups warrants careful consideration. Educational level is a recognized determinant of cognitive reserve and frailty risk in older populations. In the present study, individuals with BMS had fewer years of formal education compared to controls. Although lower education may contribute to certain vulnerability markers, particularly in cognitive screening performance, the multidimensional differences identified extended beyond cognitive domains and included objective physical performance measures (SPPB), polypharmacy rates, BMI distribution, depressive symptoms and perceived social support. The present investigation should be interpreted within its exploratory framework. It represents the first study to examine frailty in BMS using the SUNFRAIL+ model and was not designed to establish independent causal determinants. Previous studies in BMS cohorts have reported cognitive and neurofunctional alterations even after accounting for demographic variability, suggesting that the observed vulnerability profile may not be entirely attributable to educational differences alone [30, 31]. Nevertheless, residual confounding cannot be excluded, and future studies incorporating multivariate modelling will be necessary to clarify the independent contribution of educational [32].

There were high levels of frailty among the cohort with BMS. At the first‐level screening, 69.2% of BMS patients scored above the frailty threshold on the SUNFRAIL checklist, compared to only 23.1% of controls. The physical domain showed markedly higher rates of polypharmacy, defined as the concurrent use of more than five medications (76.9% vs. 30.8%, *p* < 0.001), reduced mobility (61.5% vs. 42.3%, *p* = 0.05). Surprisingly, patients with BMS reported fewer falls than controls (23.1% vs. 42.3%, *p* = 0.037), likely reflecting protective behavioural adaptations such as reduced mobility or hypervigilance. However, these adaptations may in turn promote further deconditioning and functional decline. Second‐level assessments confirmed and deepened these findings. Therapeutic adherence was generally high in both groups, as TAS scores of 3–4 indicate good adherence. Although patients showed slightly lower TAS scores (median 3 vs. 4; *p* = 0.001) and reported more frequent instances of forgetfulness or skipped doses, these differences do not suggest clinically meaningful non‐adherence. These behaviours likely reflect a multifactorial burden involving cognitive decline, emotional distress and reduced health literacy. Memory complaints, poor executive function and depressive symptoms are frequent in this population and can compromise treatment consistency and planning. As observed by Iovino et al. [15] in the SUNFRAIL+ cohort, loneliness, cognitive symptoms and poor mental quality of life were major predictors of non‐adherence, with over half reporting memory difficulties and nearly half citing forgetfulness as the primary reason for missed doses. Although suboptimal adherence was present in controls, the BMS group showed a more pronounced and systemic frailty profile affecting self‐care capacity. Improving adherence in this context requires tailored interventions integrating cognitive and emotional support, simplified therapeutic regimens and digital tools to enhance engagement and continuity of care.

Nutritional assessment with the MNA‐SF did not show significant differences between groups, indicating that overall nutritional status was comparable. However, patients with BMS demonstrated lower adherence to the Mediterranean diet (median PREDIMED score: 8 vs. 9; *p* = 0.01). Although the absolute difference was modest, it suggests a less optimal dietary pattern in the BMS group. In addition, BMI distribution differed significantly (*p* < 0.001), with BMS patients more frequently falling outside the normal weight range. In contrast, no significant differences were found in appetite loss, reduced mobility, weight loss, or calf circumference. These findings suggest that, despite similar global nutritional scores, BMS patients show greater weight variability and slightly poorer adherence to a Mediterranean dietary model [33].

Although such differences may reflect behavioural adaptations related to chronic oral discomfort or reduced food enjoyment, the cross‐sectional design precludes causal inferences. Furthermore, inflammatory and metabolic parameters were not assessed; therefore, any potential link between dietary patterns and systemic biological mechanisms remains hypothetical [34].

Overall, the findings indicate that dietary pattern and BMI distribution differ in BMS even in the absence of overt malnutrition [35]. This supports the inclusion of dietary assessment in the clinical evaluation of these patients, while further longitudinal studies are needed to clarify the clinical relevance of these differences.

Objective physical performance was significantly lower in BMS patients, with reduced SPPB total scores (median 9 vs. 12; *p* < 0.001) and poorer results across gait speed, chair stand and balance tests. Scores ≤ 9 are widely associated with reduced mobility and increased frailty risk. Despite this, BMS patients reported fewer falls than controls. As fall history was self‐reported and physical activity levels or exposure to mobility‐related situations were not assessed, this discrepancy cannot be fully interpreted within the present dataset. Importantly, frailty reflects reduced physiological reserve and functional vulnerability rather than the mere occurrence of adverse events such as falls [18]. Therefore, reduced SPPB scores should be interpreted as indicators of increased functional vulnerability, even in the absence of a higher self‐reported fall rate in this cohort. These results confirm the presence of functional decline that may not be fully captured by environmental or timed assessments alone, such as AFEAT and TUG, which showed no significant between‐group differences.

Patients with BMS showed lower scores on both the GPCOG and Qmci screening tools, with differences particularly evident in immediate recall, verbal fluency, logical memory and visuospatial performance [36]. These findings suggest reduced performance in attention‐dependent and executive domains. Cognitive performance in this context is likely multifactorial. Chronic pain is known to interfere with attentional resources and executive functioning, and medications commonly used in BMS management (e.g., anticonvulsants and antidepressants) are associated with cognitive side effects. Emotional distress and perceived sleep difficulties may further contribute to reduced cognitive efficiency. As pharmacological exposure, treatment duration and pain intensity were not adjusted for in domain‐specific analyses, it is not possible to determine whether the observed differences reflect primary neurocognitive alterations or secondary effects related to pain burden and treatment. The term “burning fog” has been proposed to describe this cognitive profile [37]; however, within the present cross‐sectional design, it should be considered a descriptive clinical construct rather than evidence of a distinct neurobiological entity.

Neuroimaging studies have provided further support, showing that individuals with BMS exhibit significantly higher Age‐Related White Matter Changes (ARWMC) scores, indicating an increased burden of white matter hyperintensities [38, 39]. These findings suggest that both vascular and degenerative mechanisms may contribute to cognitive dysfunction in BMS. In this context, recent research has identified blood‐based biomarkers such as plasma phosphorylated tau (p‐tau 217) and neurofilament light chain (NfL) as promising indicators of neurodegeneration and axonal injury [40]. Although these biomarkers have not yet been investigated in BMS, their use with neuroimaging findings including ARWMC may offer new insights into the potential links between chronic pain, aging and cognitive decline [41]. Although not yet studied in BMS, their future application could help clarify the relationship between chronic pain and cognitive vulnerability.

Depressive symptoms were significantly more frequent in the BMS group across multiple GDS‐15 items, with high prevalence of emptiness, helplessness, fear and worthlessness. The emotional profile of BMS appears distinct from typical geriatric depression, being characterized less by anhedonia and more by emptiness, fear and social withdrawal. These features may both result from and exacerbate chronic pain and functional impairment. Several studies have highlighted this bidirectional relationship.

Previous studies have reported a high prevalence of anxiety, depressive symptoms and sleep complaints in patients with BMS. Malta et al. identified anxiety as a potential predictor of BMS onset [42], while Di Bello et al. reported frequent affective disorders in middle‐aged and older adults with BMS [43, p20]. Canfora et al. found that anxiety, depression and self‐reported sleep disturbances were associated with greater pain severity and functional interference [4].

Importantly, sleep disturbances in these studies are based on subjective assessments and do not reflect objective neurophysiological sleep measures. Therefore, these findings likely represent perceived sleep difficulties, potentially influenced by psychological distress, rather than objectively confirmed primary sleep disorders [44]. Together, these data support a biopsychosocial interpretation of BMS, in which emotional distress and perceived psychosocial burden are closely associated with symptom severity. However, given the cross‐sectional design, causality cannot be established [45].

Patients with BMS reported significantly higher levels of depressive symptoms and lower perceived social support compared to controls. Differences were observed both in total GDS scores and across several specific domains, including life satisfaction, hopelessness, emptiness, reduced happiness and subjective memory complaints. In parallel, total SPS scores and multiple subdomains (Reliable Alliance, Social Integration, Attachment and Reassurance of Worth) were significantly lower in the BMS group.

These findings indicate that BMS is associated with greater emotional distress and reduced perceived relational support [46]. However, due to the cross‐sectional design, the direction of this association cannot be determined. It remains unclear whether reduced social support and depressive symptoms represent pre‐existing vulnerability factors that may increase susceptibility to BMS, or whether they emerge because of persistent oral pain and its impact on daily functioning [44].

Chronic pain conditions are known to interact bidirectionally with mood and social functioning through complex behavioural, cognitive and neurobiological mechanisms. Ongoing discomfort may lead to withdrawal, reduced participation in social activities and negative affect. Conversely, limited emotional support and pre‐existing depressive traits may influence pain perception and coping strategies. The present findings support the presence of this association in BMS but do not allow causal inferences.

Finally, QoL was significantly reduced across all subdomains of the SF‐12 in those with BMS. Both Physical and Mental Component Summaries showed marked impairment (PCS: *p* < 0.001; MCS: *p* < 0.001), with lower scores in general health, bodily pain, vitality, social functioning and role limitations. Notably, this deterioration was not limited to specific aspects but extended across all physical and emotional dimensions, including role physical, role emotional and mental health. These results confirm that the burden of BMS extends well beyond localized oral discomfort and interferes with everyday functioning and psychological well‐being. The pattern of reduced quality of life aligns with previous reports by López‐Jornet et al. [47], who observed similar trends in SF‐36 scores, and De Luca et al. [14], who found strong associations between frailty, cognitive decline and reduced quality of life using the SUNFRAIL+ framework.

Overall, the present findings support the interpretation of BMS within a biopsychosocial framework. From a biological standpoint, patients showed differences in BMI distribution and lower adherence to the Mediterranean diet, despite comparable global nutritional status. Psychologically, BMS patients reported significantly higher levels of depressive symptoms, emotional distress and perceived sleep difficulties. Socially, they described lower perceived support, particularly in relational domains such as attachment, reassurance of worth and social integration, while financial vulnerability did not differ between groups.

Although these dimensions are analytically distinct, they are likely interconnected through complex and potentially bidirectional mechanisms. Emotional distress may influence pain perception and coping strategies; persistent pain may contribute to social withdrawal and mood changes; and reduced social support may weaken resilience.

**FIGURE 4:**
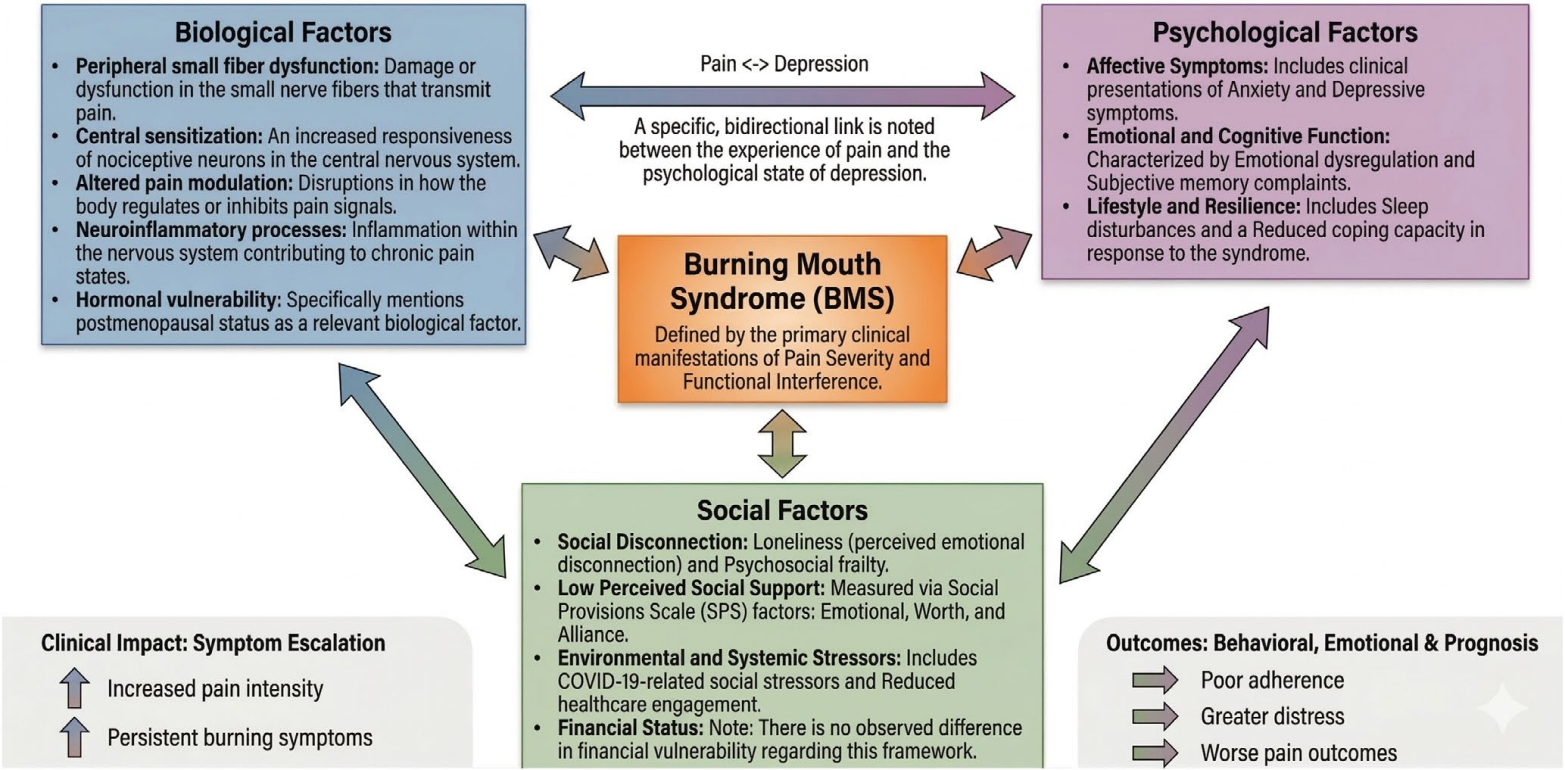
The biopsychosocial conceptual framework of burning mouth syndrome (BMS).

Taken together, the convergence of biological variability, psychological burden and relational vulnerability suggests that BMS should not be viewed solely as an isolated oral disorder. Rather, it appears embedded within a broader multidimensional context that may influence symptom expression and overall well‐being (**Figure** **4**).

Innovative approaches, such as the Blueprint Persona model and ICT‐based tools [48], may help address fragmentation in care and support long‐term patient engagement.

## 5 Limitations

This study has several limitations. Its cross‐sectional and observational nature prevents causal inference and raises the possibility of reverse causality between BMS and frailty domains. Participants were recruited from the Oral Medicine Units of two tertiary referral centres, and the control group included individuals attending the same institutions for unrelated reasons. This convenience sampling may limit generalizability and introduce selection bias.

Despite matching for age and sex, the two groups differed in educational level. Given the established relationship between education, socioeconomic status and frailty, this difference may have influenced certain outcomes, particularly cognitive measures. Although vulnerability differences were observed across multiple biological, psychological and social domains, residual confounding related to educational attainment cannot be excluded. The study was designed as an exploratory multidimensional assessment rather than a causal modelling investigation. Future longitudinal studies should incorporate multivariate adjustment models to further clarify independent associations.

Residual confounding from unmeasured variables such as medication use, comorbidities and physical activity cannot be excluded. Several outcomes relied on self‐reported instruments (GDS, TAS, PREDIMED, SPS, SF‐12), which are inherently prone to recall and social‐desirability bias. In particular, the brevity of the TAS may lead to under‐ or overestimation of medication adherence. Cognitive performance measures (GPCOG and Q*mci* screen) may also have been influenced by factors such as pain intensity, depressive mood, sleep disturbances, or polypharmacy, which were not adjusted for during test administration. Moreover, the study did not include biological or imaging biomarkers, which could have strengthened the multidimensional assessment by providing objective indicators of systemic and neurocognitive vulnerability. The study was adequately powered for medium‐to‐large effects but may have been underpowered for small effect sizes, which should therefore be interpreted with caution. Lastly, the lack of longitudinal data prevents evaluation of frailty progression or treatment effects over time.

## 6 Conclusions

The present findings indicate that BMS, traditionally viewed as a localized chronic oral pain disorder, is associated with higher levels of frailty across multiple frailty domains in this older cohort of patients (≥ 65 years) in Italy. The results suggest that those with BMS would benefit from detailed comprehensive geriatric and frailty assessment to tailor interventions to reduce future frailty‐related adverse outcomes including risk of hospitalization, institutionalization, cognitive decline and advancing frailty. Further, long‐term follow up of this cohort is planned to show the differential long‐term consequences for patients with BMS. In particular, older adults with BMS show a vulnerability profile involving physical, cognitive, emotional, social and nutritional domains. These results highlight the importance of routine frailty screening, using tools such as SUNFRAIL+, along with integrated, multidisciplinary management. Beyond pharmacological treatment, care should include psychological support, lifestyle and nutritional counselling and social engagement strategies to promote early prevention and personalized interventions. Optimizing treatment adherence remains a clinical priority. Addressing cognitive and motivational barriers, including forgetfulness and emotional burden, alongside the implementation of digital health technologies, may enhance patient engagement and continuity of care. In conclusion, recognizing BMS as a condition associated with multidimensional frailty supports a paradigm shift in both research and clinical practice, promoting comprehensive, person‐centred care and interdisciplinary collaboration aimed at improving functional outcomes and QoL for these older patients.

## Author Contributions

**Federica Canfora:** conceptualization; methodology; investigation; data curation; writing – original draft; writing – review and editing. **Giulia Ottaviani:** methodology; investigation; writing – review and editing. **Antonietta Argiuolo:** formal analysis; statistical analysis; validation. **Alessandra Giorgiutti:** investigation; data curation; resources. **Guido Iaccarino:** data curation; validation. **Katia Rupel:** investigation; writing – review and editing. **Sabina Saccomanno:** methodology; validation. **Vincenzo De Luca:** investigation; resources. **Michele Virgolesi:** investigation; data curation. **Elizabeth Moloney:** writing – review and editing; validation. **Rónán O'Caoimh:** writing – review and editing; Supervision. **Giuseppe Liotta:** project administration; supervision. **Michele Davide Mignogna:** supervision; funding acquisition. **Maddalena Illario:** project administration; supervision. **Daniela Adamo:** conceptualization; methodology; supervision; writing – review and editing.

## Funding

The authors have nothing to report.

## Conflicts of Interest

The authors declare no conflicts of interest.

## Supporting information


**Appendix S1:** joor70191‐sup‐0001‐Supinfo.docx.

## Data Availability

The data that support the findings of this study are available from the corresponding author upon reasonable request.
