# Supplementary material for: Uncovering Frailty in Burning Mouth Syndrome: Insights From a SUNFRAIL+ Based Multidimensional Assessment
Source: J Oral Rehabil. 2026 Mar 26;53(7):1347–62. doi: 10.1111/joor.70191 (PMC13261785; doi:10.1111/joor.70191)

# SUNFRAIL QUESTIONNAIRE

Date and Place: ________________________

## PROFESSIONALS

| ☐ Nurse | ☐ GPs | ☐ Social workers |
| --- | --- | --- |
| ☐ Community actors | ☐ Other professionals | ☐ Caregiver |

## BENEFICIARIES

| Gender ☐ M ☐ F | Age ☐ 65–74 ☐ 75–85 | Level of education ☐ Low (without studies, Primary school) ☐ Medium (Secondary school or vocational degree) ☐ High (University, Master or PhD degree) |
| --- | --- | --- |
|  |  |  |

## QUESTIONS

| No. | Question | Response (☐ Yes / ☐ No) |
| --- | --- | --- |
| 1 | Do you regularly take 5 or more medication per day? | ☐ Yes ☐ No |
| 2 | Have you recently lost weight such that your clothing has become looser? | ☐ Yes ☐ No |
| 3 | Your physical state made you walking less during the last year? | ☐ Yes ☐ No |
| 4 | Have you been evaluated by your GP during the last year? | ☐ Yes ☐ No |
| 5 | Have you fallen 1 or more times during the last year? | ☐ Yes ☐ No |
| 6 | Have you experienced memory decline during the last year? | ☐ Yes ☐ No |
| 7 | Do you feel lonely most of the time? | ☐ Yes ☐ No |
| 8 | In case of need, can you count on someone close to you? | ☐ Yes ☐ No |
| 9 | Have you had any financial difficulties in facing dental care and health care cost during the last year? | ☐ Yes ☐ No |

**TAS SCALE**

|  | YES (0) | NO (1) |
| --- | --- | --- |
| Have you ever forgotten to take your medications? |  |  |
| When you feel better, do you sometimes stop taking your medications? |  |  |
| Do you sometimes skip a dose of medication? |  |  |
| Do you sometimes reduce the dosage prescribed by your doctor? |  |  |

Score interpretation: 0–2 = non-adherent; 3–4 = adherent.

**MEDITERRANEAN DIET ADHERENCE QUESTIONNAIRE (PREDIMED)**

| No. | Question | Frequency / Response (criteria for 1 point) |
| --- | --- | --- |
| 1 | Do you use olive oil as the principal source of fat for cooking? | Yes |
| 2 | How much olive oil do you consume per day (including that used in frying, salads, meals eaten away from home, etc.)? | ≥4 Tbsp |
| 3 | How many servings of vegetables do you consume per day? (Count garnish and side servings as ½ point; a full serving is 200 g.) | ≥2 |
| 4 | How many pieces of fruit (including fresh-squeezed juice) do you consume per day? | ≥3 |
| 5 | How many servings of red meat, hamburger, or sausages do you consume per day? (A full serving is 100–150 g.) | <1 |
| 6 | How many servings (12 g) of butter, margarine, or cream do you consume per day? | <1 |
| 7 | How many carbonated and/or sugar-sweetened beverages do you consume per day? | <1 |
| 8 | Do you drink wine? How much do you consume per week? | ≥7 cups |
| 9 | How many servings (150 g) of pulses do you consume per week? | ≥3 |
| 10 | How many servings of fish/seafood do you consume per week? (100–150 g of fish, 4–5 pieces or 200 g of seafood) | ≥3 |
| 11 | How many times do you consume commercial (not homemade) pastry such as cookies or cake per week? | <2 |
| 12 | How many times do you consume nuts per week? (1 serving = 30 g) | ≥3 |
| 13 | Do you prefer to eat chicken, turkey or rabbit instead of beef, pork, hamburgers, or sausages? | Yes |
| 14 | How many times per week do you consume boiled vegetables, pasta, rice, or other dishes with a sauce of tomato, garlic, onion, or leeks sautéed in olive oil? | ≥2 |

Scoring: Each question meeting the criterion = 1 point. Higher scores indicate greater adherence to the Mediterranean diet.

# Mini Nutritional Assessment (MNA®) – Screening Form

Last name: ____________________ First name: ____________________

Sex: __________ Age: __________ Weight (kg): __________ Height (cm): __________

Complete the screening by filling in the boxes with the appropriate numbers. Total the numbers for the final screening score.

## Screening

|  |  |
| --- | --- |
| A. Has food intake declined over the past 3 months due to loss of appetite, digestive problems, chewing or swallowing difficulties? | 0 = severe decrease in food intake 1 = moderate decrease in food intake 2 = no decrease in food intake |
| B. Weight loss during the last 3 months | 0 = weight loss greater than 3 kg (6.6 lbs) 1 = does not know 2 = weight loss between 1 and 3 kg (2.2 and 6.6 lbs) 3 = no weight loss |
| C. Mobility | 0 = bed or chair bound 1 = able to get out of bed/chair but does not go out 2 = goes out |
| D. Has suffered psychological stress or acute disease in the past 3 months? | 0 = yes 2 = no |
| E. Neuropsychological problems | 0 = severe dementia or depression 1 = mild dementia 2 = no psychological problems |
| F1. Body Mass Index (BMI = weight in kg / height in m²) | 0 = BMI less than 19 1 = BMI 19 to less than 21 2 = BMI 21 to less than 23 3 = BMI 23 or greater |
| F2. Calf circumference (CC) in cm (if BMI not available) | 0 = CC less than 31 3 = CC 31 or greater |

### Screening score (max. 14 points)

0–7 points: Malnourished
8–11 points: At risk of malnutrition
12–14 points: Normal nutritional status

**Short Physical Performance Battery (SPPB)**

**1. Balance Tests**

| Test | Instructions | Scoring |
| --- | --- | --- |
| Side-by-Side Stand | Feet together side-by-side for 10 seconds | <10 sec = 0 pt 10 sec = 1 pt |
| Semi-Tandem Stand | Heel of one foot against side of big toe of the other for 10 seconds | <10 sec = 0 pt 10 sec = +1 pt |
| Tandem Stand | Feet aligned heel to toe for 10 seconds | 10 sec = +2 pt 3–9.99 sec = +1 pt <3 sec = +0 pt |

**2. Gait Speed Test**

Measures the time required to walk 4 meters at a normal pace (use the best of 2 trials).

| Time (seconds) | Points |
| --- | --- |
| <4.82 sec | 4 pt |
| 4.82–6.20 sec | 3 pt |
| 6.21–8.70 sec | 2 pt |
| >8.7 sec | 1 pt |
| Unable | 0 pt |

**3. Chair Stand Test**

Pre-test: Participants fold their arms across their chest and try to stand up once from a chair.

If unable → Stop (0 pt)

If able → proceed to the test below.

5 repeats: Measure the time required to perform five rises from a chair to an upright position as fast as possible without using the arms.

| Time (seconds) | Points |
| --- | --- |
| ≤11.19 sec | 4 pt |
| 11.20–13.69 sec | 3 pt |
| 13.70–16.69 sec | 2 pt |
| >16.7 sec | 1 pt |
| >60 sec or unable | 0 pt |

**Total SPPB Score**

Sum of all sections (0–12 points). Higher scores indicate better lower extremity function.

**GENERAL PRACTITIONER (GP) VISITING CHECKLIST**

| **Question** | **Answer** |
| --- | --- |
| Do you have difficulty making an appointment with your GP? |  |
| Do you have transportation difficulties reaching your GP’s office? |  |
| In the past three months, how many times have you visited your GP? |  |
| In the past three months, have you visited the nursing clinic or received home nursing visits? |  |
| In the past three months, how many hospitalizations have you had? |  |
| Reason for hospitalization: |  |
| After hospital discharge, did you contact your GP? |  |
| How many emergency room visits have you had in the past three months? |  |
| Reason for the emergency room visit: |  |
| After the emergency room visit, did you contact your GP? |  |
| Have you had specialist visits, blood tests, or other diagnostic exams in the past three months? |  |
| After the specialist visits and/or diagnostic exams, did you contact your GP? |  |

**Age-Friendly Environmental Assessment Tool (AFEAT)**

| Question | Response (0–4 scale) |
| --- | --- |
| I am able to access local services such as shops, restaurants, maintenance services, or GP clinics without difficulty. | Strongly disagree (0) Disagree (1) Neither agree nor disagree (2) Agree (3) Strongly agree (4) |
| I am able to move around my local area/community without difficulty. | Strongly disagree (0) Disagree (1) Neither agree nor disagree (2) Agree (3) Strongly agree (4) |
| I live close to local services such as shops, restaurants, maintenance services, and medical clinics. | Strongly disagree (0) Disagree (1) Neither agree nor disagree (2) Agree (3) Strongly agree (4) |
| My housing is safe, clean, and well-maintained. | Strongly disagree (0) Disagree (1) Neither agree nor disagree (2) Agree (3) Strongly agree (4) |
| There are many places to meet with friends and family and to take part in community activities. | Strongly disagree (0) Disagree (1) Neither agree nor disagree (2) Agree (3) Strongly agree (4) |
| There is ongoing outreach to include people at risk of social isolation. | Strongly disagree (0) Disagree (1) Neither agree nor disagree (2) Agree (3) Strongly agree (4) |
| I feel that I am a valued part of my local community. | Strongly disagree (0) Disagree (1) Neither agree nor disagree (2) Agree (3) Strongly agree (4) |
| I can engage in voluntary or paid activities without worrying about special needs. | Strongly disagree (0) Disagree (1) Neither agree nor disagree (2) Agree (3) Strongly agree (4) |
| I have easy access to information about the local community and can express my views in community decision-making. | Strongly disagree (0) Disagree (1) Neither agree nor disagree (2) Agree (3) Strongly agree (4) |
| I have easy access to information and services about my health. | Strongly disagree (0) Disagree (1) Neither agree nor disagree (2) Agree (3) Strongly agree (4) |

# Quick Mild Cognitive Impairment Screen (Qmci)


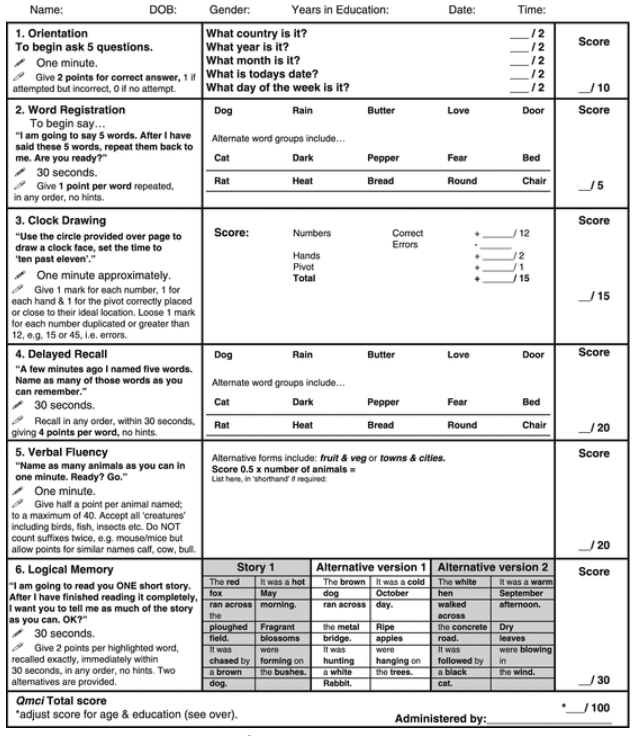


**GENERAL PRACTITIONER ASSESSMENT OF COGNITION (GPCog)**


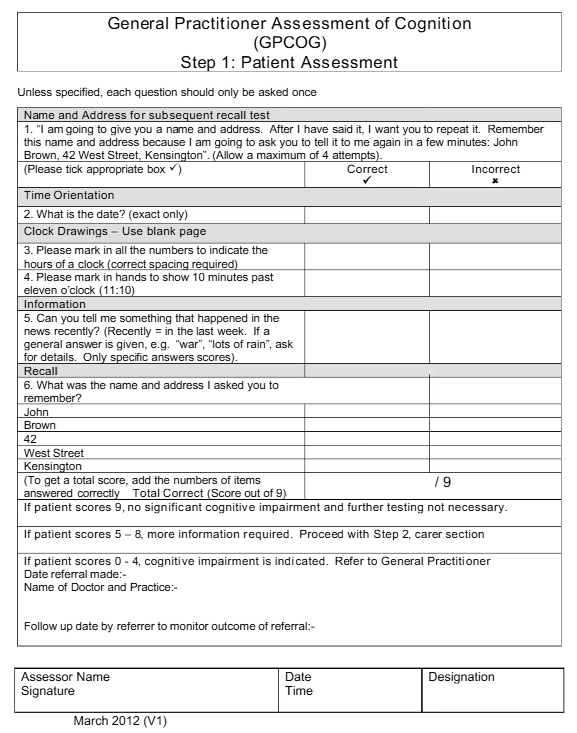


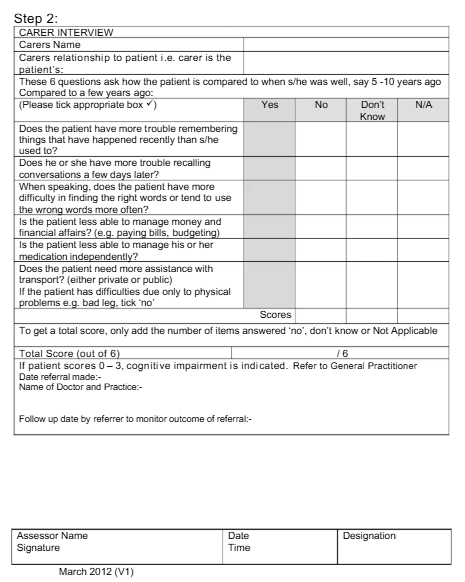


**GERIATRIC DEPRESSION SCALE (GDS)**

| ***Question*** | ***Yes*** | ***No*** |
| --- | --- | --- |
| Are you basically satisfied with your life? | Yes (0) | No (1) |
| Have you dropped many of your activities and interests? | Yes (1) | No (0) |
| Do you feel that your life is empty? | Yes (1) | No (0) |
| Do you often get bored? | Yes (1) | No (0) |
| Are you in good spirits most of the time? | Yes (0) | No (1) |
| Are you afraid that something bad is going to happen to you? | Yes (1) | No (0) |
| Do you feel happy most of the time? | Yes (0) | No (1) |
| Do you often feel helpless? | Yes (1) | No (0) |
| Do you prefer to stay at home rather than going out and doing new things? | Yes (1) | No (0) |
| Do you feel you have more memory problems than most people? | Yes (1) | No (0) |
| Do you think it is wonderful to be alive now? | Yes (0) | No (1) |
| Do you feel pretty worthless the way you are now? | Yes (1) | No (0) |
| Do you feel full of energy? | Yes (0) | No (1) |
| Do you think your situation is hopeless? | Yes (1) | No (0) |
| Do you think that most people are better off than you are? | Yes (1) | No (0) |

**SCORING**: Normal: 3 ± 2; Mildly depressed: 7 ± 3; Severely depressed: 12 ± 2

**SOCIAL PROVISIONS SCALE (SPS)**

| ***Question*** | ***Strongly Disagree*** | ***Disagree*** | ***Agree*** | ***Strongly Agree*** |
| --- | --- | --- | --- | --- |
| There are people I can count on to help me in case of real need (material help) | 1 | 2 | 3 | 4 |
| There are people who enjoy the same social activities as I do (social integration) | 1 | 2 | 3 | 4 |
| I feel part of a group of people who share my attitudes and beliefs (social integration) | 1 | 2 | 3 | 4 |
| There are close people who give me a sense of emotional security and well-being (attachment) | 1 | 2 | 3 | 4 |
| There is someone with whom I can discuss important decisions about my life (guidance) | 1 | 2 | 3 | 4 |
| I have relationships in which my competence and professionalism are recognized (self-affirmation) | 1 | 2 | 3 | 4 |
| There is a reliable person to whom I can turn in case of problems (guidance) | 1 | 2 | 3 | 4 |
| I have a strong emotional bond with at least one other person (attachment) | 1 | 2 | 3 | 4 |
| There are people who admire my talents and abilities (self-affirmation) | 1 | 2 | 3 | 4 |
| There are people I can count on in case of emergency (material help) | 1 | 2 | 3 | 4 |

**Self-Assessment Questionnaire on Socio-Economic Conditions**

| ***Category*** | ***Options*** |
| --- | --- |
| Perceived financial status / income sufficiency | Sometimes has difficulty making ends meet Barely sufficient to get by Has more than needed to live comfortably No answer / missing |
| Annual household income | Less than €10,000 €10,000–€15,000 €15,001–€30,000 €30,001–€50,000 More than €50,000 Missing / Don’t know / Prefer not to say |
| Self-rated health | Poor Fair Good Very good Excellent |
| Number of chronic diseases | None 1 condition 2–3 conditions ≥4 conditions |
| Citizenship | Italian EU citizen Non-EU citizen |
| Marital status | Married / In a couple Divorced / Separated Widowed Never married Other / unspecified |
| Education level | Below high school diploma High school diploma / institute Above high school diploma |
| Registered with a general practitioner | No Yes |
| Household size | 1 2 3 4 ≥5 |


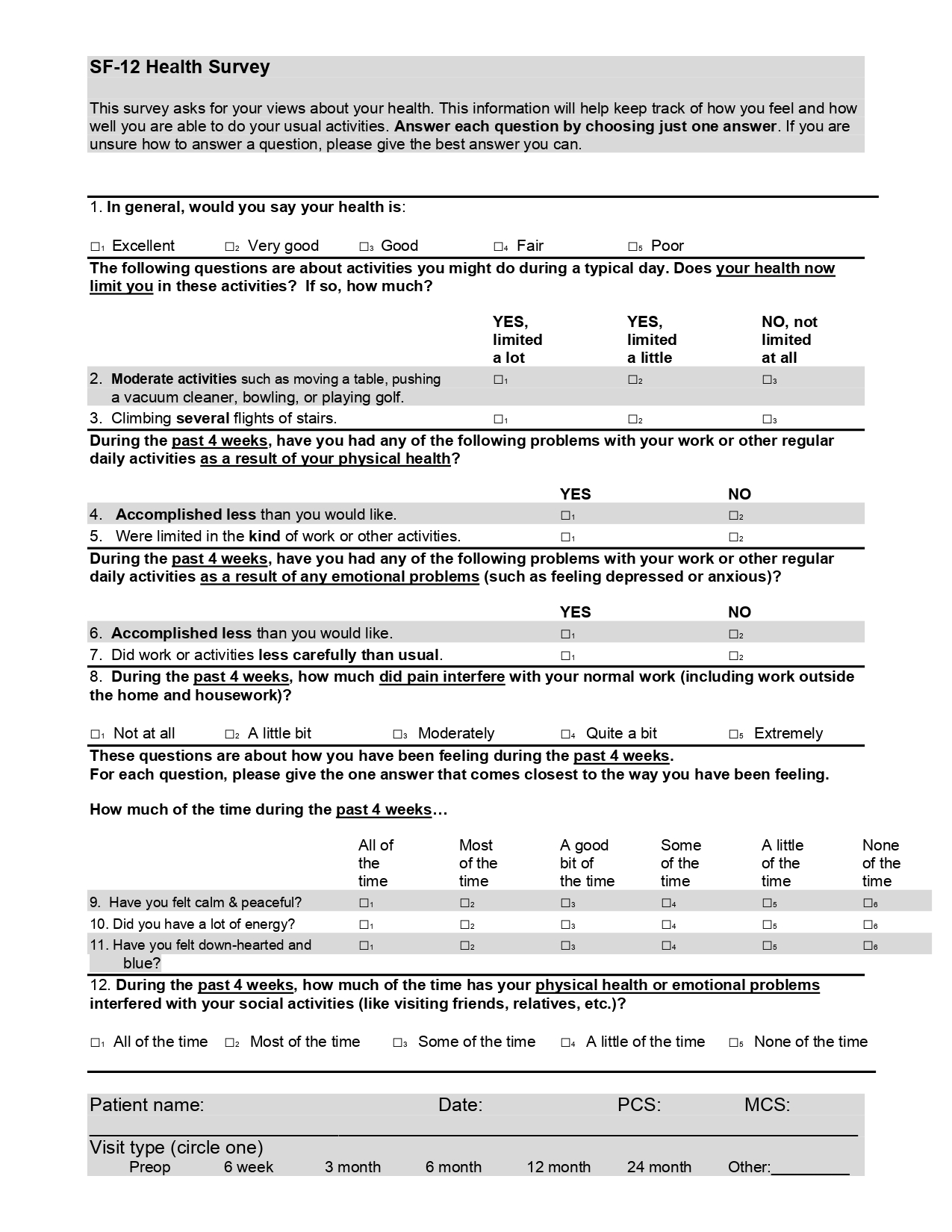

Supplement: Supplementary file 1 — Appendix S1: joor70191‐sup‐0001‐Supinfo.docx. [file JOOR-53-1347-s001.docx]
